# Comparative analysis of the chrysanthemum transcriptome with DNA methylation inhibitors treatment and silencing MET1 lines

**DOI:** 10.1186/s12870-023-04036-x

**Published:** 2023-01-21

**Authors:** Dongru Kang, Muhammad Ayoub Khan, Pan Song, Yvru Liu, Yifei Wu, Penghui Ai, Zhongai Li, Zicheng Wang

**Affiliations:** State Key Laboratory of Crop Stress Adaptation and Improvement, Plant Germplasm Resources and Genetic Laboratory, Kaifeng Key Laboratory of Chrysanthemum Biology, School of Life Sciences, Henan University. Jinming Road, Kaifeng, 475004 Henan China

**Keywords:** Curcumin, 5-Azacytidine, Flower induction, Gene expression, DNA methylation

## Abstract

**Background:**

As one of the ten most famous flowers in China, the chrysanthemum has rich germplasm with a variety of flowering induction pathways, most of which are photoperiod-induced. After treatment with DNA methylation inhibitors, it was found that DNA methylation plays an important role in flowering regulation, but the mechanism of action remains unclear. Therefore, in this study, curcumin, 5-azaC, their mixed treatment, and MET1_-RNAi_ lines were used for transcriptome sequencing to find out how different treatments affected gene expression in chrysanthemums at different stages of flowering.

**Results:**

Genomic DNA methylation levels were measured using HPLC technology. The methylation level of the whole genome in the vegetative growth stage was higher than that in the flowering stage. The methylation level of DNA in the vegetative growth stage was the lowest in the curcumin and mixed treatment, and the methylation level of DNA in the transgenic line, mixed treatment, and curcumin treatment was the lowest in the flowering stage. The flowering rate of mixed treatment and curcumin treatment was the lowest. Analysis of differentially expressed genes in transcriptomes showed that 5-azaC treatment had the most differentially expressed genes, followed by curcumin and transgenic lines, and mixed treatment had the fewest. In addition, 5-azaC treatment resulted in the differential expression of multiple DNA methylation transferases, which led to the differential expression of many genes. Analysis of differentially expressed genes in different treatments revealed that different treatments had gene specificity. However, the down-regulated GO pathway in all 4 treatments was involved in the negative regulation of the reproductive process, and post-embryonic development, and regulation of flower development. Several genes associated with DNA methylation and flowering regulation showed differential expression in response to various treatments.

**Conclusions:**

Both DNA methylase reagent treatment and targeted silencing of the *MET1* gene can cause differential expression of the genes. The operation of the exogenous application is simple, but the affected genes are exceedingly diverse and untargeted. Therefore, it is possible to construct populations with DNA methylation phenotypic diversity and to screen genes for DNA methylation regulation.

**Supplementary Information:**

The online version contains supplementary material available at 10.1186/s12870-023-04036-x.

## Background

After more than 1,600 years of cultivation and breeding, the chrysanthemum has evolved into numerous varieties [1], and the total number of existing chrysanthemum varieties are now more than 30,000. Chrysanthemum cultivars have a variety of petal types, such as flat, spoon-shaped, and tubular ray florets [2]. Summer, autumn, and winter are the natural flowering season of the chrysanthemum. Among all chrysanthemum types, the autumn chrysanthemum type is the most common, so most chrysanthemum varieties are short-day plants that respond to the photoperiod to induce flowering. The regulation of DNA methylation in photoperiod-induced flowering is a scientific area worth researching, particularly in the chrysanthemum, which can provide genetic resources to improve flowering based on an understanding of the flowering mechanism.

In higher plants, there are many unsolved problems in the regulatory mechanism of DNA methylation [3]. Scientists applied exogenous DNA methylase inhibitors to treat plants [4–7], expecting to obtain differential phenotypes and further analyze their possible epigenetic mechanisms. 5-Azacytidine(5-azaC) is the most common demethylation agent due to its structural similarity to cytosine. For instance, in *Arabidopsis thaliana*, lowered methylation levels caused by 5-azaC can take the place of low temperatures in stimulating plant flowering [8], and this effect has been proven in numerous species using analogous techniques [9].Our research group's previously discovered that a low concentration of 5-azaC can improve the tillering ability of wheat while also affecting the heading, flowering date, 1000-grain weight, and other indicators, whereas a high concentration of 5-azaC can inhibit the growth of wheat roots and seedlings. When studying chrysanthemums, it was also found that 5-azaC treatment would mainly cause changes in the flowering period [10, 11], and variations in leaf morphology, flower morphology, and flower characteristics were prevalent [12, 13]. Through statistical analysis of the flowering induction time and flowering development time of chrysanthemum varieties, it was found that lengthening or shortening of the flowering induction time among different lines resulted in different flowering times for the treated lines [10, 12]. In conclusion, 5-azaC treatment resulted in a variety of phenotypic changes in the chrysanthemum. However, the effect of 5-azaC treatment on gene expression in chrysanthemums has not been investigated.

Curcumin has been used extensively in cancer research as an anti-mutant and anti-cancer agent, recently found to induce epigenetic changes [14]. Curcumin can upregulate miR-29b expression levels, resulting in *DNMT3b* silencing and loss of phosphatase and tensin homolog methylation.[15]. Curcumin gives rise to promoter hypomethylation and enhanced *FANCF* gene (Fanconi anemia group F protein) expression levels in the SiHa cell line [16]. Curcumin significantly represses Egr-1 expression at both transcriptional and translational levels by reducing the DNA-binding activity of the transcription factor Egr-1 towards curcumin-responsive elements [17]. Toll-like receptor-4 gene expression is suppressed by curcumin, which inhibits NF-B [17]. Curcumin can induce *HSP70* gene expression by initially depleting intracellular Ca^2+^, followed by repression of p53 gene function in the target cells [18]. Different treatments of curcumin, such as tissue culture [19] or soaking cuttings [20] in an aqueous solution, can alter the flowering stage of the chrysanthemum. Treatment can lead to a reduction in genome-wide DNA methylation levels in chrysanthemums [21], but the impact of curcumin on gene expression in chrysanthemums has not been studied.

In chrysanthemum flower induction, DNA methylase-associated genes' expression patterns revealed that *CmMET1*(methyltransferase 1) and *CmCMT3*(methyltransferase 3) exhibited a downward trend while *CmDRM2* (domains rearranged methyltransferase 2) showed an up-regulation trend, suggesting that different DNA methylase gene functions played different roles in flowering induction [10]. When the *CmMET1* gene was silenced, the flowering period of the chrysanthemum variety ‘Zijingling’ was advanced, and the scion could be influenced by grafting [11]. Transcriptome analysis revealed that the silenced *CmMET1* gene can cause differential expression of many genes at the young stage, although the DEGs of the *MET1-*_*RNAi*_ line in the flowering stage are not clear. Furthermore, it is not clear which genes are affected by treatment with 5-azaC and curcumin to regulate flowering in chrysanthemums. Therefore, the effect of treatment on the gene expression of chrysanthemums needs to be explored. Transcriptome analysis is an efficient method to obtain differentially expressed genes, and the analysis of the difference between multiple groups combined with transgenic strains can provide clues for subsequent studies.

## Results

### Chrysanthemum phenotype after DNA methylase inhibitor treatment

**Fig. 1:**
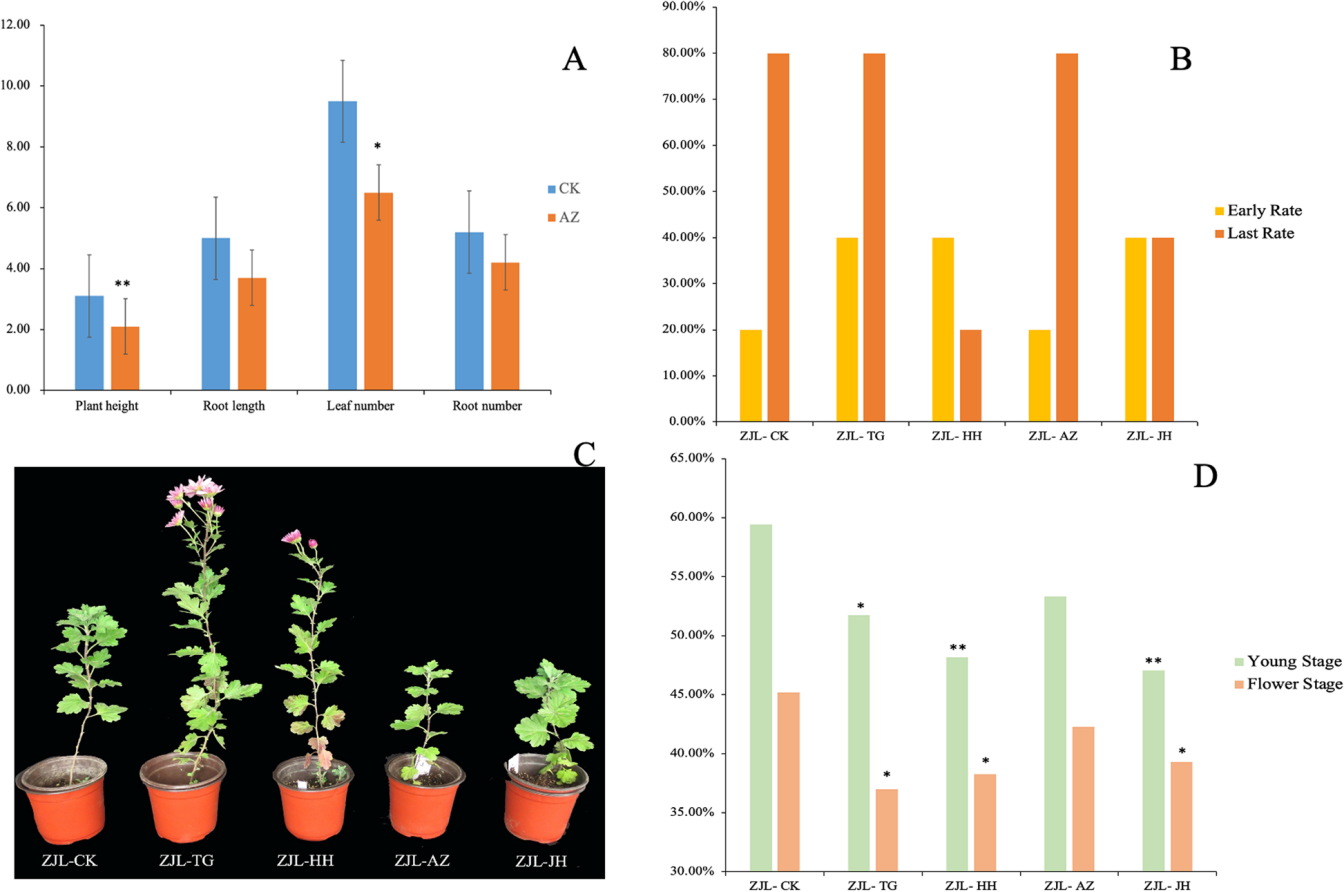
**A**. Plant growth data statistics of ‘Zijingling’ treated with 5-azaC (30 d). **B** The budding rate of the two stages under different treatments. **C** Different treatments for flowering phenotypes. **D** Different treatment levels of DNA methylation. Note: CK was the control group, AZ was the 5-azaC treatment group, JH was the curcumin treatment group, HH was the 5-azaC mixed with the curcumin treatment group, and TG was the MET1-RNAi transgenic lines. ZJL is short for Zijingling. ‘*’ means significant difference, ‘**’ means highly significant difference, *P* < 0.05

After 30 days of tissue culture treatment, the growth data of the transplanted material were statistically analyzed (Fig.1-A). It was found that the plant height and number of leaves in the 5-azaC treatment group were significantly less than those in the control group. The total number of roots and root length in the treatment group showed a non-significant reduction compared to the control group.

Statistical analysis of early and late flowering rates of tissue culture-treated materials after transplantion declared that transgenic lines, mixed treatment, and curcumin treatment had the earliest flowering; the early flowering rate was slightly higher than that of control and 5-azaC treatment (Fig. 1-B). In the late flowering stage, the flowering rate was higher in the transgenic line and 5-azaC treatment than in the mixed treatment and curcumin treatment. Mixed treatment and curcumin treatment could decrease the flowering rate of the chrysanthemum.

However, through the flowering time of ‘ZJL’, it was found that TG treatment had the earliest flowering followed by mixed treatment, while curcumin and 5-azaC treatments were comparable, but all the groups already had budded (Fig. 1-C). The characteristic of 5-azaC treated lines is that they can bud and blossom at a low growth rate, thus flowered earlier than the control. The growth and flowering times of the transgenic lines were higher than those of the control lines.

### Effects of multiple treatments on genome-wide DNA methylation levels

Genome-wide methylation levels were measured in leaves at the young and flowering stages of 5 groups of materials (Fig. 1-D). When compared to the control group at a young stage, DNA methylation level in TG lines was significantly lower, AZ samples showed marginally lower level, while HH and JH treatments showed highly significantly lower level. The methylation levels of all treated genomic DNA lines decreased; however, the reduction in AZ-treated lines was less than the reduction in other treatment lines. The intensity of methylation in the flowering stage was further reduced compared with that in the young stage, and the degree of methylation in AZ, JH, HH, and TG treatments compared with the control lines was 2.92%, 5.93%, 6.95%, and 8.21%, respectively, in which JH, TG, and HH had significant differences compared to the control. However, the difference between each treatment and the control decreased., This may be due to the fact that, after a prolong period of growth recovery following treatment, the effect of the methylation treatment agent gradually decreased as a result of certain self-regulation of plants, which reduce the difference between the treatment and the control lines.

According to the reduction degree of DNA methylation level, the reduction effect of DNA methylation level in chrysanthemum treated with curcumin and 5-azaC (mixed) did not dominating effect over a single methylation inhibitor, and their combination may not have a superposition effect on the reduction of DNA methylation level in chrysanthemum.

Additionally, we used transgenic lines (ZJL-_Met_) to conduct curcumin, 5-azaC, and mixed treatments (Table 1). Further application of exogenous DNA methylase inhibitors to transgenic lines will lead to further reduction of the genomic DNA methylation level, with TG&HH treatment having the largest reduction, followed by TG&JH treatment and TG&AZ treatment.

**Table 1:** Comparison of DNA methylation levels between treatment and control lines in chrysanthemum (HPLC)

Cultivars	Abbs	Treatment	Concentration (μmol L^-1^)	DNA methylation levels	
				Young Stage	Flower Stage
ZJL	CK	-	-	59.44	45.22
ZJL	AZ	5-azaC	100	53.32^a^	42.30
ZJL	JH	Curcumin	200	47.09**	39.29^a^
ZJL	HH	5-azaC & Curcumin	100&200	48.18**	38.27^a^
ZJL-_Met1_	TG	-	-	51.73	37.01^a^
ZJL-_Met1_	TG&AZ	5-azaC	100	46.83^a^	-
ZJL-_Met1_	TG&JH	Curcumin	200	45.38^a^	-
ZJL-_Met1_	TG&HH	5-azaC & Curcumin	100&200	44.47^a^	-

^a^ indicates the significance of the difference at the level of 0.05

^**^ indicates the significance of the difference at the level of 0.01

ZJL-_Met1_ transgenic plants with normal chrysanthemum, lower than the level of methylation after dealing with the 5 azaC and curcumin, further reduce the degree of methylation, but less methylation loss, presumably has a certain degree of methylation falling within the scope of the plant itself has the certain regulation ability, can't unlimited.

### Analyze the effects of various treatments on the gene expression

#### De novo assembly of sequence reads using Illumina sequencing

Three biological replicate samples of AZ, JH, HH, TG, and CK were obtained. This allowed us to determine the impact of various treatments on the transcriptomic alteration in "Zijingling." A total of 681,917,276 raw reads and 680,962,980 clean reads were produced via reference transcriptome sequencing (Table S2). *C. nankingense* genome was used as a reference genome. Considering that chrysanthemum was only a proximal plant, the transcriptome comparison rate of 15 samples ranged from 37.95%-63.89% (Figure S1).

**Fig. 2:**
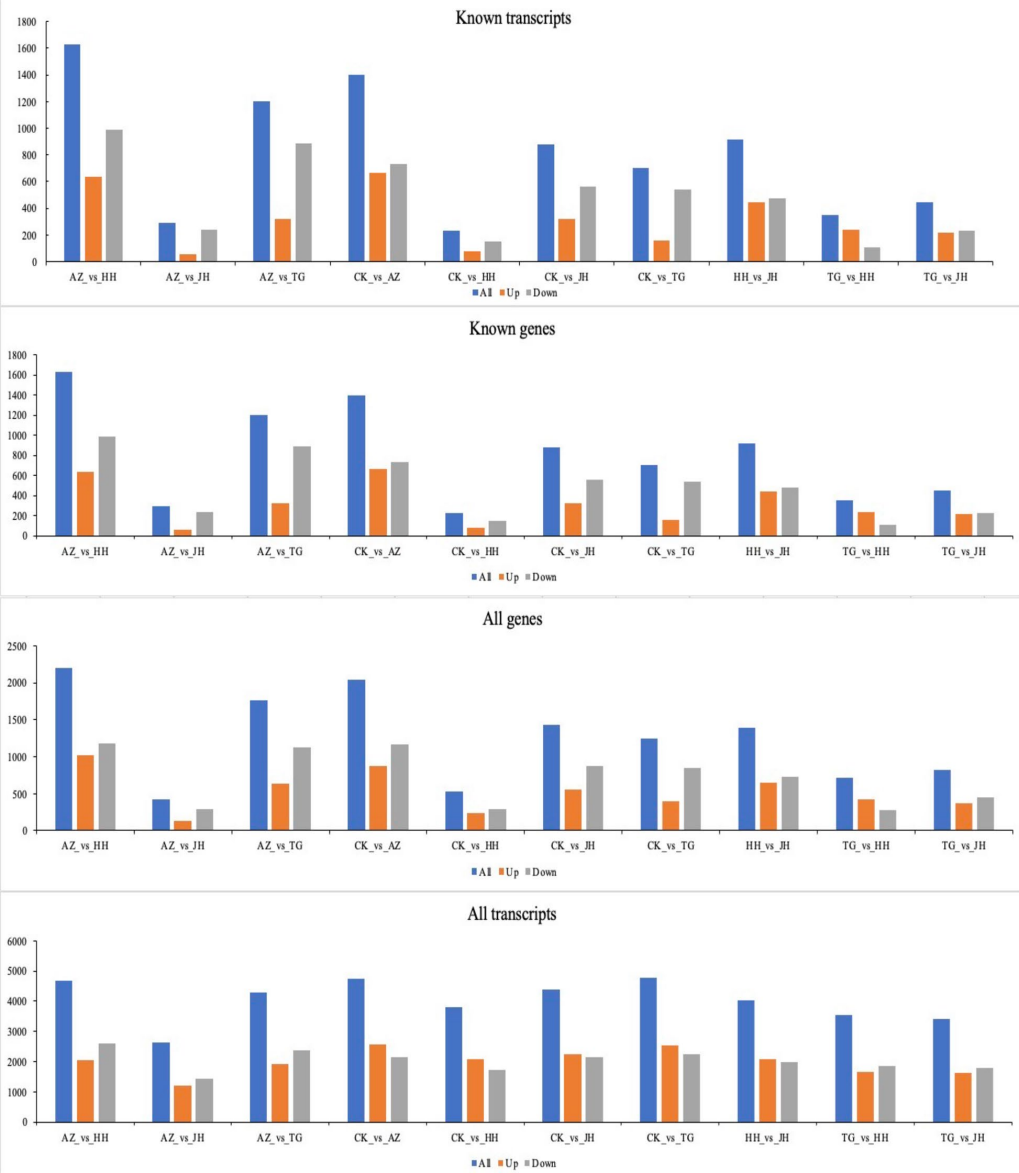
Differentially expressed genes and transcripts were compared in all samples. Note: The vertical axis represents the number of known genes, known transcripts, all genes, and all transcripts

After assembling all clean readings, a total of 179412 transcripts with an average length of 1122 bp were obtained. After all clean reads were assembled from 15 samples and Intergenic transcript. A complete match of the intron chain and a potentially novel isoform accounted for 90.93% of the total (Fig. 2).

#### Gene’s functional annotation and classification

To obtain the putative annotations, the assembled uniqueness was aligned to the following six databases: NR, NT, Swiss-Prot, KEGG, KOG, GO, and InterPro (Table S2). Overall, 178913 genes were successfully annotated using all six databases. A total of 54068 (30.22%) genes were annotated using the NR database. In total, 36551 genes were matched in the Pfam database, which is about 20.43% of all annotated genes (Table S2). The Swiss-Prot database matched 34398 genes, which is about 19.23% of all annotated genes (Table S2). NR database annotations were highest, followed by Pfam, GO and Swiss-Prot database annotations, KEGG annotation results were less, while the lowest results were found in String annotation.

There were 44257 genes mapped into 134 KEGG pathways which can be divided into 6 large pathways including “cellular processes”, “environmental information processing”, “genetic information processing”, “human diseases”, “metabolism”, and “organismal systems”.

#### Screening and analysis of DEGs

The gene expression histogram of all 15 samples indicated that the overall gene expression level was similar (Fig. 2). All the transcripts showed a close number of DEGs, but the number of DEGs was decreased in all the genes, with variations among different treatments. The deviation was more obvious in known genes and transcripts, the lowest number of DEGs was between mixed treatment and control while the highest number was recorded between AZ treatment and HH treatment. The down-regulated genes were more than up-regulated genes in all the samples.

**Fig. 3:**
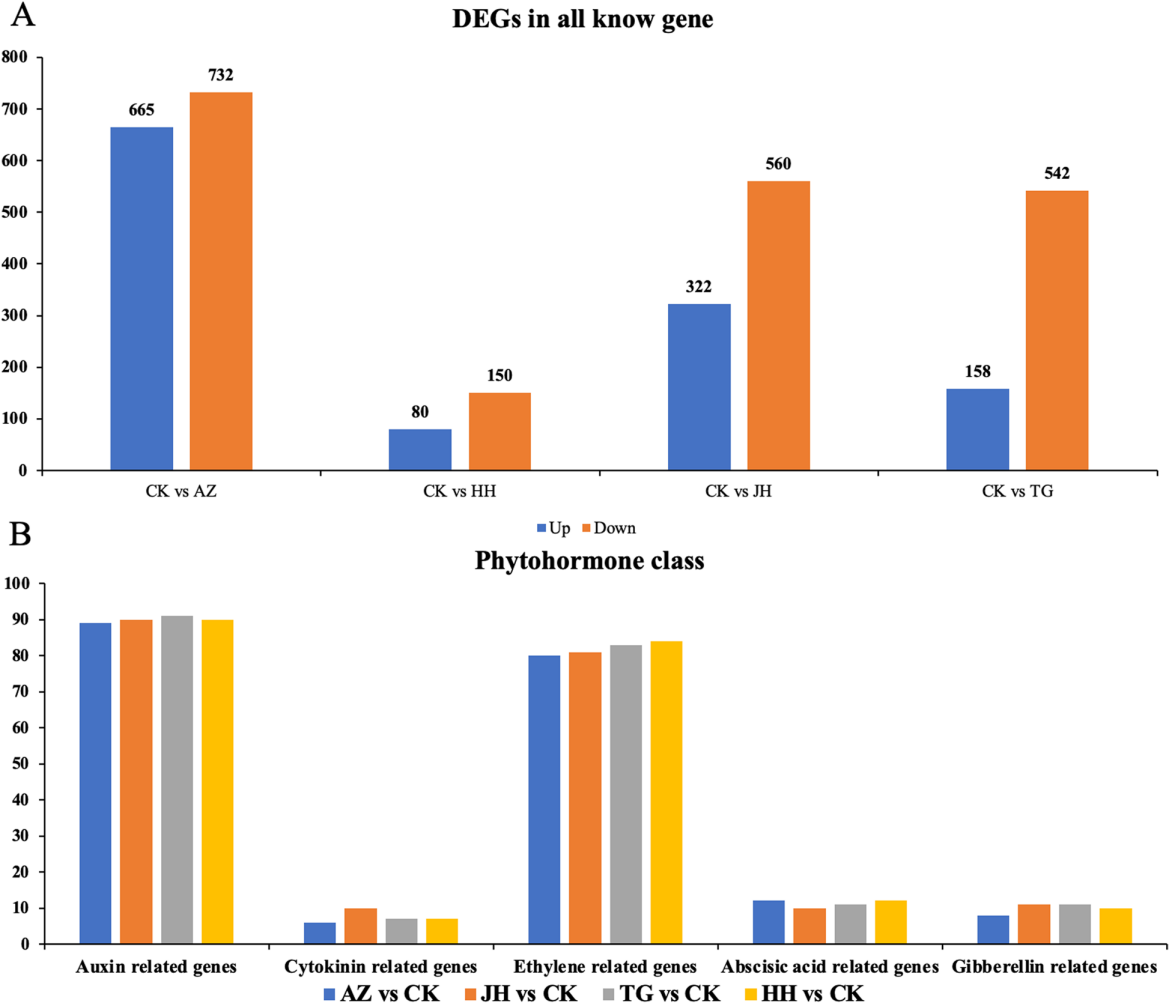
The number of DEGs in the known genes in the 4 treated samples was compared with the control samples. Note: A. The gene number of differentially expressed genes (DEGs). B. The gene number of five plant hormone-related genes in DEGs

We conducted a thorough comparative analysis of the DEGs (*P*-value ≤0.01 and |log2Ratio| ≥ 1) in four comparisons including CK vs AZ, CK vs JH, CK vs TG and CK vs HH. In the CK vs AZ comparison, there were 732 genes that were down-regulated while 665 were up-regulated. Meanwhile, 150 genes were down-regulated, and 80 genes were up-regulated in the CK vs HH combination. In the CK vs TG comparison, there were 542 and 158 genes, down-regulated and up-regulated, respectively. Meanwhile, 560 and 322 genes were down-regulated and up-regulated in the CK vs JH combination, respectively. Overall, there were more down-regulated genes than up-regulated genes between different treatments (Fig.3-A). According to the analysis of the results of plant hormone-related genes in DEGs, it was found that there was no significant difference in the number of five major hormone-related genes among different treatments (Fig.3-B).

#### GO, KEGG, and Ipath pathways analysis of DEGs

In this study, GO analyses were performed to categorize the activities of the annotated DEGs under various DNA methyltransferase inhibitors treated and transgenic lines (Table 2). The "metabolic process," "cellular process," and "single-organism process" were the most significantly enriched GO terms in the biological processes across all comparisons.

**Table 2:** The top ten most enriched GO terms of DEGs in all comparison groups GO term hierarchy

**GO term hierarchy 1**	**GO term** **hierarchy 2**	**Number of DEGs**							
		**CKvsAZ**		**CKvsJH**		**CKvsHH**		**CKvsTG**	
		Up	Down	Up	Down	Up	Down	Up	Down
Biological process	cellular process	294	372	30	64	135	262	71	245
	metabolic process	277	337	33	56	141	231	66	193
	response to stimulus	158	209	15	40	74	184	29	178
Cellular component	cell	334	467	42	82	162	313	79	301
	cell part	333	464	42	82	162	307	76	300
	organelle	226	364	27	59	122	198	53	213
	membrane	181	256	20	34	82	182	40	147
	membrane part	145	188	17	20	66	141	33	118
Molecular function	binding	282	372	35	74	133	297	74	268
	catalytic activity	300	316	41	64	136	262	53	209

Metabolic pathways had the most genes, followed by “biosynthesis of secondary metabolites”, “biosynthesis of antibiotics”, and “Toxoplasmosis” in the CKvsAZ, CKvsHH, and CKvsTG groups. However, in the CKvsJH group, the pathways with the highest numbers of genes were “Glycolysis / Gluconeogenesis”, followed by “Pentose phosphate pathway”, “Pentose and glucuronate interconversions”, and “Fructose and mannose metabolism”. The supplemental Table contains information on the top 30 and top 10 pathways (Table S3 and Table S4). According to the results of pathway decomposition, the pathways affected by 5-azC treatment and curcumin treatment were different, but 5-azaC treatment was similar to the transgenic line (MET1_-RNAi_).

**Fig. 4:**
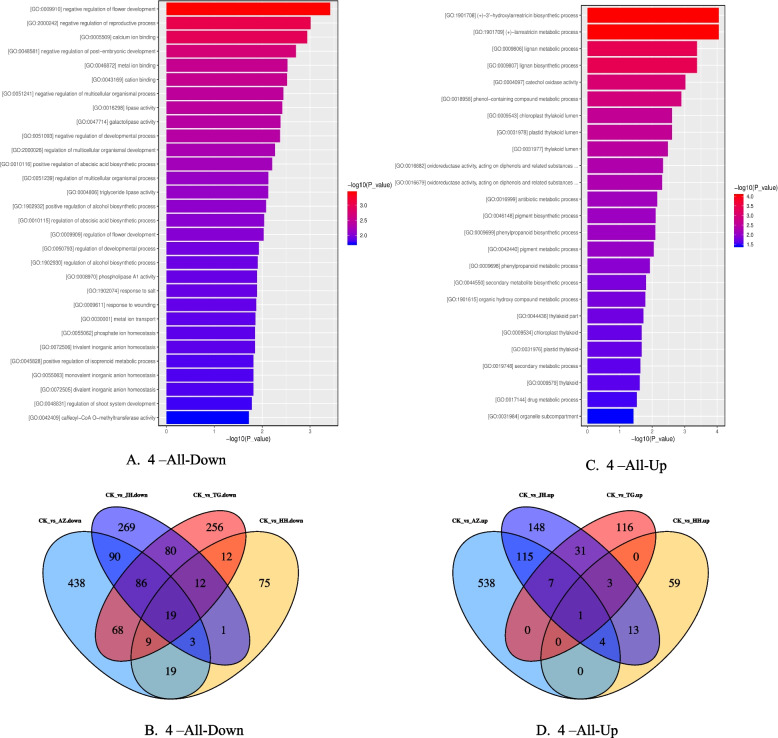
Venn diagram analysis of differentially expressed genes between different treatments. Note: A. KEGG pathway results were down-regulated in all the 4 groups (CKvsAZ, CKvsJH, CKvsTG, CKvsHH). B. Venn diagrams of down-regulated genes in all 4 groups C. Venn diagrams of up-regulated genes in all 4 groups. D. KEGG pathway results were up-regulated in all the 4 groups

The GO and KEGG pathway analyses were carried out on the DEGs of the 5 groups of samples (Table 3). Among them, special attention was paid to genes that were differentially expressed in the four treatments. The results showed that the genes up regulated in the four samples were mostly secondary metabolites and other ways. For example: (+)-3'-hydroxylarreatricin biosynthetic process, (+)-larreatricin metabolic process, lignan metabolic process and lignan biosynthetic process. The downregulation of DNA methylation can lead to changes in multiple secondary metabolite pathways. It is very noteworthy that genes downregulated in the four treatments involve multiple GO pathways related to flower development (Fig.4). For example, negative regulation of flower development, negative regulation of the reproductive process, negative regulation of post-embryonic development, and regulation of flower development. In addition, the DNA methyltransferase activity is also inhibited.

**Table 3:** The top five most enriched KEGG of DEGs in all comparison groups

Code	Enriched KEGG Pathway of DEGs			
	CKvsAZ	CKvsJH	CKvsHH	CKvsTG
1	Ribosome	Biosynthesis of antibiotics	Cell cycle	Alcoholism
2	Photosynthesis	Phenylpropanoid biosynthesis	Aminoacyl-tRNA biosynthesis	MAPK signaling pathway-plant
3	Biosynthesis of antibiotics	Carbon metabolism	Starch and sucrose metabolism	Cell cycle
4	Carbon metabolism	Alcoholism	DNA replication	Carbon metabolism
5	Photosynthesis - antenna proteins	Insulin signaling pathway	Meiosis – yeast	DNA replication

Therefore, each the four treatments will cause changes in the flowering period of chrysanthemums. The evidence suggests that the flower development of the chrysanthemum is closely related to DNA methylation.

**Fig. 5:**
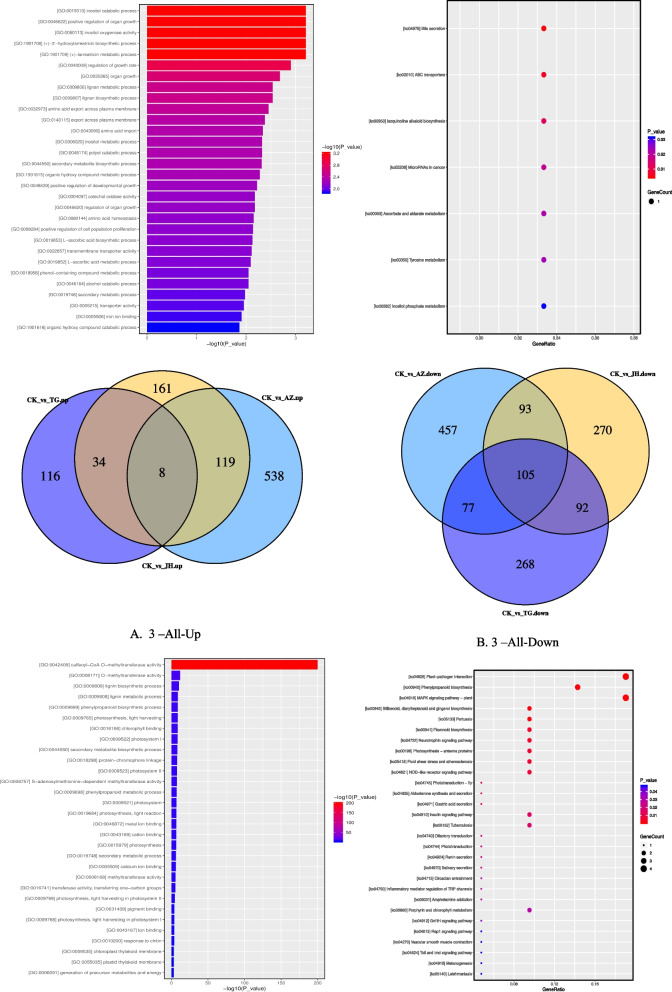
Venn diagram analysis of differentially expressed genes between different treatments. Note: A. Venn diagrams of up-regulated genes in all 3 groups (CKvsAZ, CKvsJH, CKvsTG), GO and KEGG pathway results B. Venn diagrams of down-regulated genes in all 3 groups, GO and KEGG pathway results

Considering the complexity of mixed treatment, we only carried out GO pathway analysis of DEGs in JH, AZ, and TG 3 treatments and found that the up-regulated pathways in all 3 treatments were mostly secondary metabolite synthesis pathways (Fig. 5), such as inositol catabolic process, positive regulation of organ growth, inositol oxygenase activity,(+)-3'-hydroxylarreatricin biosynthetic process,(+)-larreatricin metabolic process, regulation of growth rate, organ growth, and lignan metabolic process. All down-regulated genes involved methyltransferase, lignin metabolic pathways, and photosynthesis-related pathways, such as caffeoyl-CoA O-methyltransferase activity, O-methyltransferase activity, lignin biosynthetic process, lignin metabolic process, phenylpropanoid biosynthetic process, photosynthesis, light harvesting, chlorophyll-binding, and photosystem I.

**Fig. 6:**
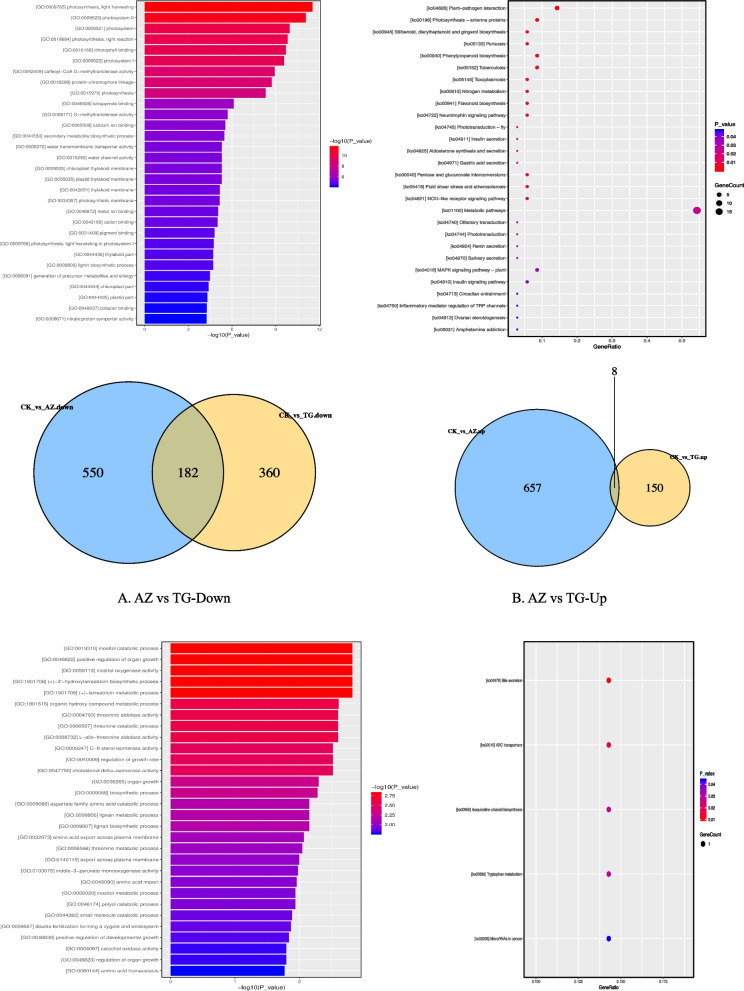
Venn diagram analysis of differentially expressed genes between AZ and TG. Note: A. Venn diagrams of up-regulated genes in 2 groups (CKvsAZ, CKvsTG), GO and KEGG pathway results B. Venn diagrams of down-regulated genes in 2 groups, GO and KEGG pathway results

As a DNA methyltransferase inhibitor, 5-azaC inhibits the DNMT gene. After silencing the MET1 gene, it can be seen that AZ will cause a greater number of DEGs, while the effect range of silencing the MET1 gene is smaller and more targeted (Fig. 6). Through comparative analysis of DEGs between these two samples, we found that the GO pathways in both AZ and TG were up-regulated as follows: inositol catabolic process, positive regulation of organ growth, inositol oxygenase activity, (+)-3'-hydroxylarreatricin biosynthetic process, (+)-larreatricin metabolic process, organic hydroxy compound metabolic process, and threonine aldolase activity. The down-regulated genes in both AZ and TG treatment were enriched in the GO pathway, which was mainly a photosynthesis-related pathway, for example: photosynthesis, light harvesting, photosystem II, photosynthesis, light reaction, chlorophyll binding, photosystem I.

**Fig. 7:**
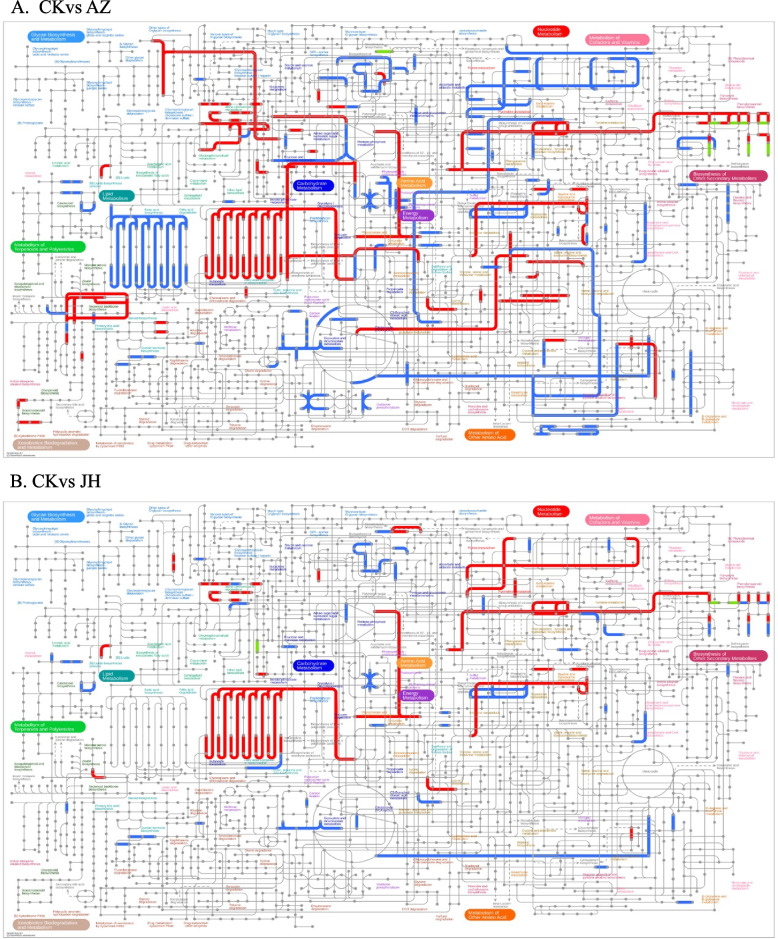

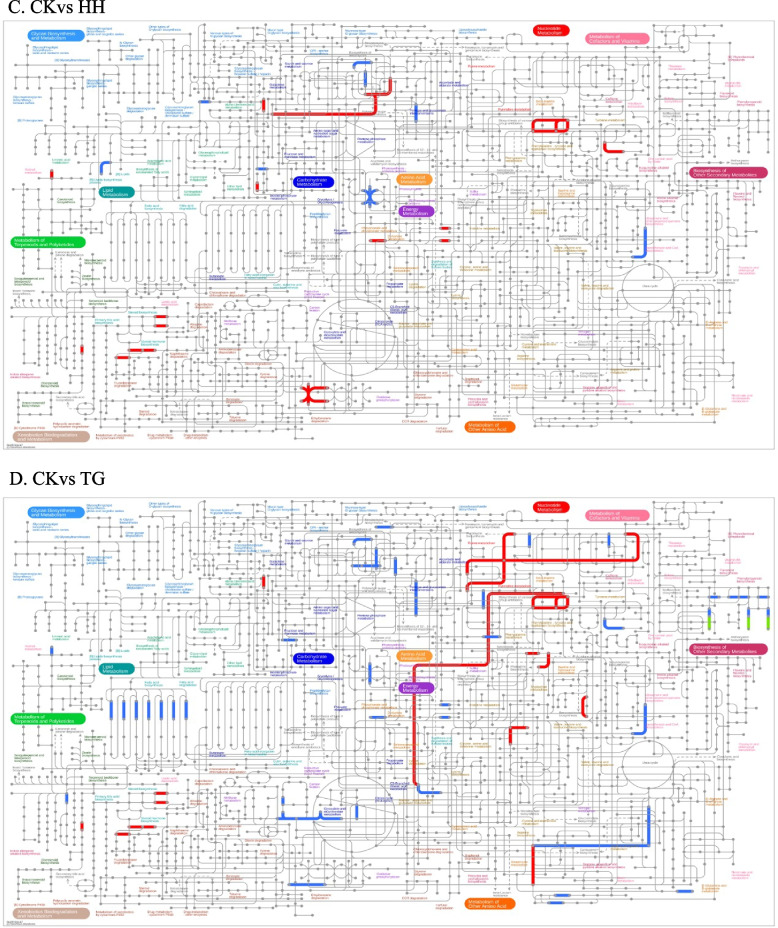
The IPATH pathway analysis results between 4 groups of samples and control. Note: The red line represents the pathway affected by up-regulated genes, the blue line represents the pathway affected by down-regulated genes, and the yellow line represents the pathway affected by both up-regulated and down-regulated genes. IPATH analysis is divided into four modules: Metabolic, secondary, antibiotic, and metabolic

Based on the pathway results analyzed by IPATH, 5-azaC treatment has the greatest effect on chrysanthemum, which can cause up-regulation and down-regulation expression of multiple pathways (Fig. 7). The mixed treatment had the least effect on the gene, and the effect of the curcumin treatment was greater than that of MET silenced strain. It can be concluded that the exogenous application of single DNA methylase inhibitors has the greatest effect on contemporary genes in plants.

#### Identification of DEGs involved in the DNA methylation and flower-related genes

**Fig. 8:**
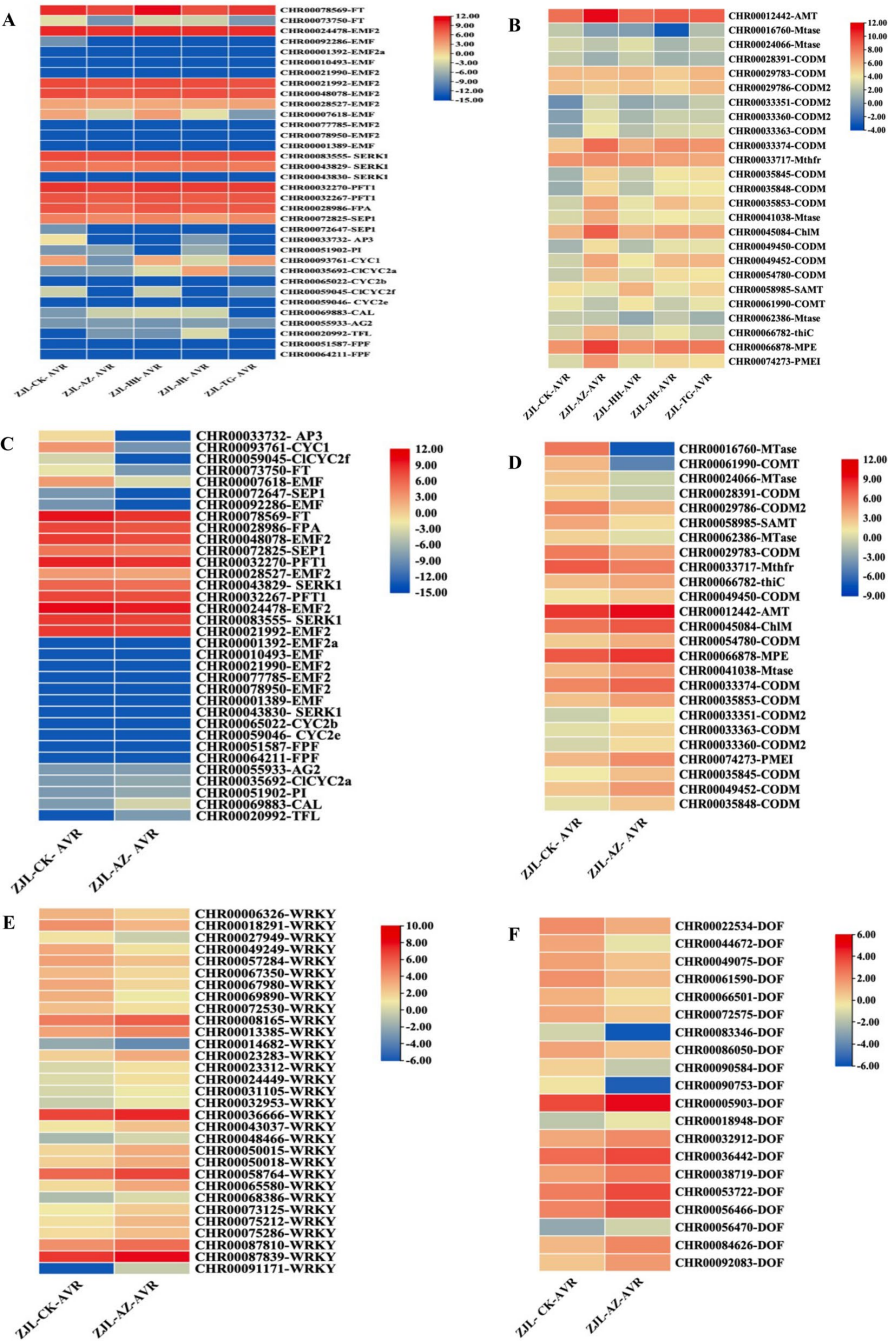
Heat map of the selected genes. (**A**) Genes associated with DNA methylation in all treatments. (**B**) Genes associated with flowering induction in all treatments. (**C**) Genes associated with DNA methylation in all treatments. (**D**) Genes associated with flowering induction in AZ treatment. (**E**) Analysis of the selected genes in the WRKY transcription factor family. (**F**) Analysis of the selected genes in the DOF transcription factor family

Considering that curcumin and 5-azaC (mixed) and *MET1* silencing are both related to DNA methylation in the genome. We extracted DNA methylation-related genes in the DEGs and analyzed their expression levels with heat maps (Fig. 8). In addition, the phenotypes of our treated lines showed differences in flowering time, so we also focused on some genes related to flowering and analyzed the expression levels of those genes.

Only AZ treatment of the *AMT* gene leads to up-regulation of expression. *Mtase* gene was down-regulated in the treatment of AZ, JH and HH, the down-regulation was most in JH, while TG is not affected. For DNA methylation genes, the expression of 15 unigenes was up-regulated, and the expression of 9 unigenes was down-regulated. The expression of *EMF* (embryonic flower) was downregulated in all treatments. The *EMF* gene is affected by all treatments in varying degrees of down-regulation except the HH treatment, which did not affect this gene. The expression of the *FT* gene decreased in TG and AZ treatment, *TFL* was unaffected by TG, and the expression of other treatments is upregulated. *CAL* gene is only up-regulated in AZ, JH, and HH, and down-regulated in TG. The expression of CYC2a and HH, and JH was upregulated, and the others were not affected. The expression of *CYC2f* HH treatment did not affect, and the other treatments all caused down-regulation, with TG having less effect. *CYC1* genes were all down-regulated in the AZ and JH, the rest did not affect, PI was up-regulated in the AZ and JH, and HH and TG were down-regulated. Seven unigenes were down-regulated, five unigenes were up-regulated, and the remaining 22 genes had smaller expression folds.

The analysis of the WRKY transcription factor family found that 31 members showed significant differential expression in AZ treatment, among them, nine were up-regulated and 22 were down-regulated; 20 members of the DOF transcription factor family were also affected, of which, 10 were up-regulated and 10 were down-regulated. The effects of this treatment were different for different members, and the functions of specific members need further exploration and research.

#### Identification of DEGs of transcription factors (TFs)

Many TFs changed dramatically under different DNA methyltransferase inhibitors in treated and transgenic lines, so we enhanced the screening criteria. The TFs with a |log2Ratio| ≥ 2 in each comparison were examined further. The top 12 transcription factor families with DEGs were selected for histogram drawing.

**Fig. 9:**
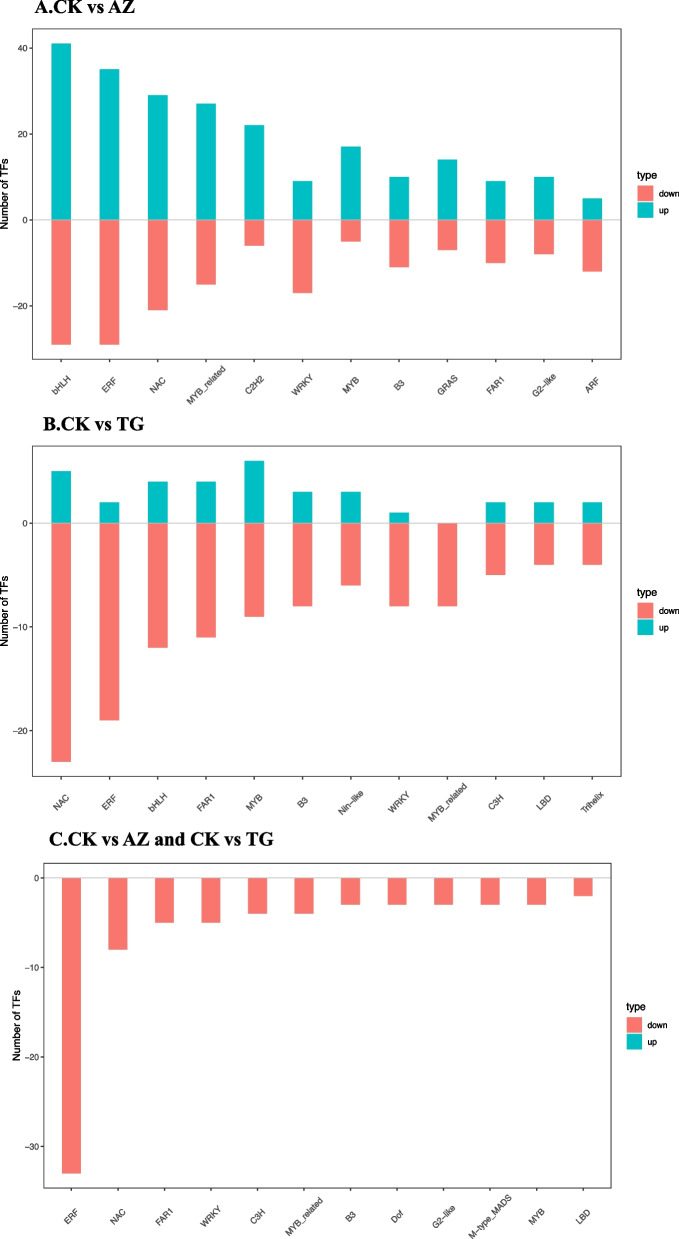

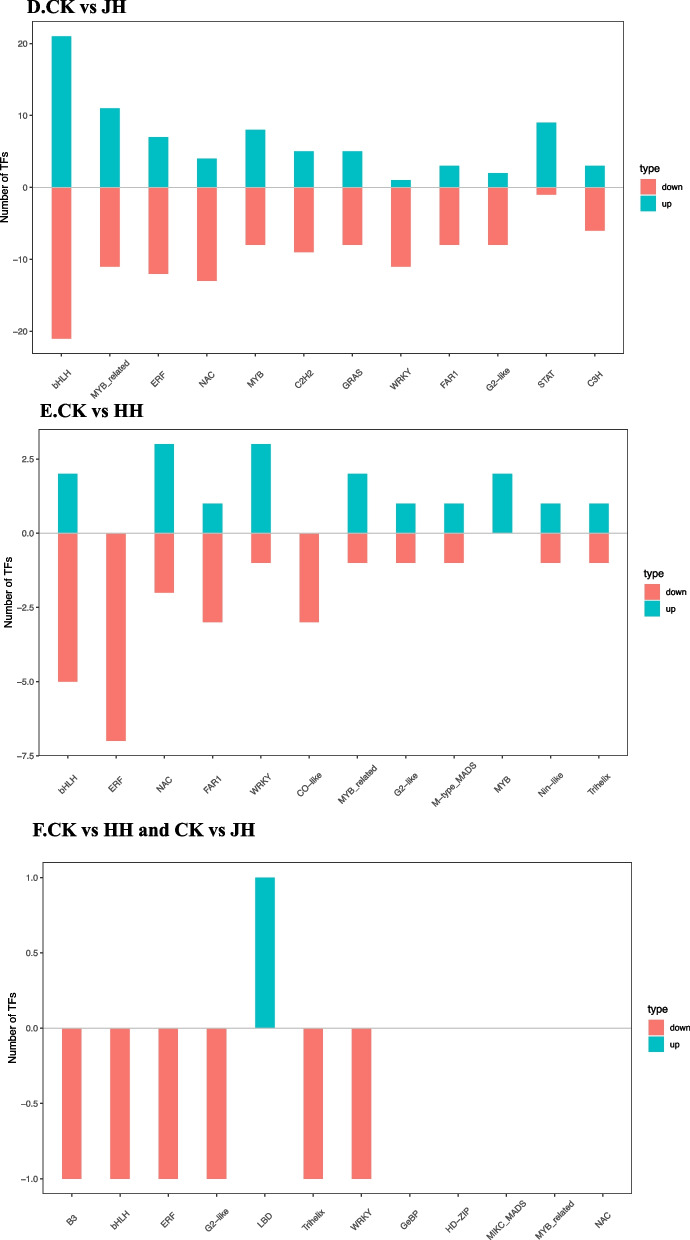
Histogram analysis of differentially expressed transcription factor families. (**A**) Control and AZ treatment were compared (**B**) Control and TG lines were compared (**C**) Analysis of differentially expressed transcription factors between AZ treatment and transgene (**D**) Control and JH treatment were compared (**E**) Control and HH treatment were compared (**F**) Analysis of differentially expressed transcription factors between JH treatment and mixed treatment (HH treatment)

Among all the treatments, compared with the control, there were 1379 genes belonging to 48 gene families in the AZ treatment, 882 DEGs belonged to 46 transcription factor families in the JH treatment, 700 DEGs belonged to 44 families in transgenic lines, and only 230 genes belonged to 34 families in the HH treatment (Fig. 9). The highest number of genes are still impacted most significantly by the AZ treatment.AZ treatment resulted in the differential expression of several genes in the bHLH, ERF, NAC, MYB, C2H2, and WRKY gene families, and the number of up-regulated expressions was greater than the number of down-regulated expressions. Compared with the control, the number of down-regulated genes was much higher than that of up-regulated genes, and the transcription factor families included bHLH, ERF, NAC, MYB, C2H2, and WRKY. The majority of the DEGs were determined to be downregulated after further comparison.

The SNP point mutation analysis of 15 samples showed that all the point mutations were mainly A-G, C-T, G-A and T-C4, among which CK had the highest number of mutations. The SNP mutation sites were mainly in the Exon, followed by the inter-gene region and intra-gene region (Figure S2). There were few SNP mutations in the 5 'UTR' 3 'UTR region, and upstream and downstream sequences. InDel mutation sites were similar to SNP sites, among which the CK mutation frequency was higher than that of various treatments and transgenic lines (Figure S3).

#### Variable shear analysis

Genome-wide changes in DNA methylation status were induced by curcumin and 5-azaC treatment, as well as *MET1* silencing. It has been established that DNA methylation in the promoter region is closely related to gene expression. However, it is not clear whether the altered intragenic DNA methylation state will affect variable shearing. Therefore, we conducted a comparative analysis of variable shearing between the treatment group and the control group.

**Fig. 10:**
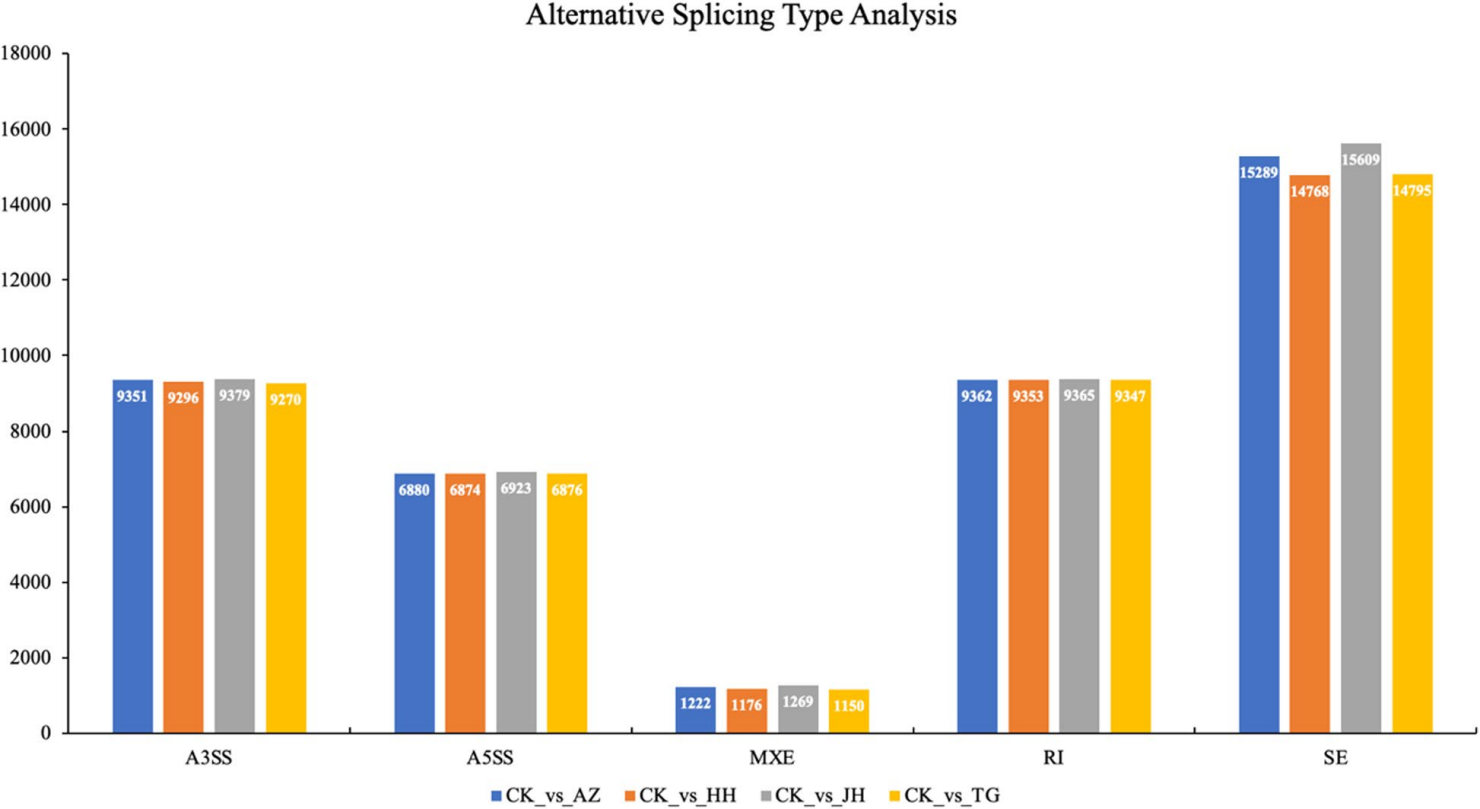
Five types of alternative splicing were compared and analyzed in 4 groups of samples. Note A3SS (Alternative 3’splice site); A5SS (Alternative 5’splice site); MXE (Mutually exclusion exon); RI (Retained intron).SE (Skipped exon)

According to the statistical results, the types and quantities of variable shear were basically the same between the 4 different samples and the control. Among different alternative shear types, SE was the highest, followed by RI and A35S, and while A53S was the least followed by MXE (Fig. 10).

#### qRT-PCR validation of differentially expressed genes

**Fig. 11:**
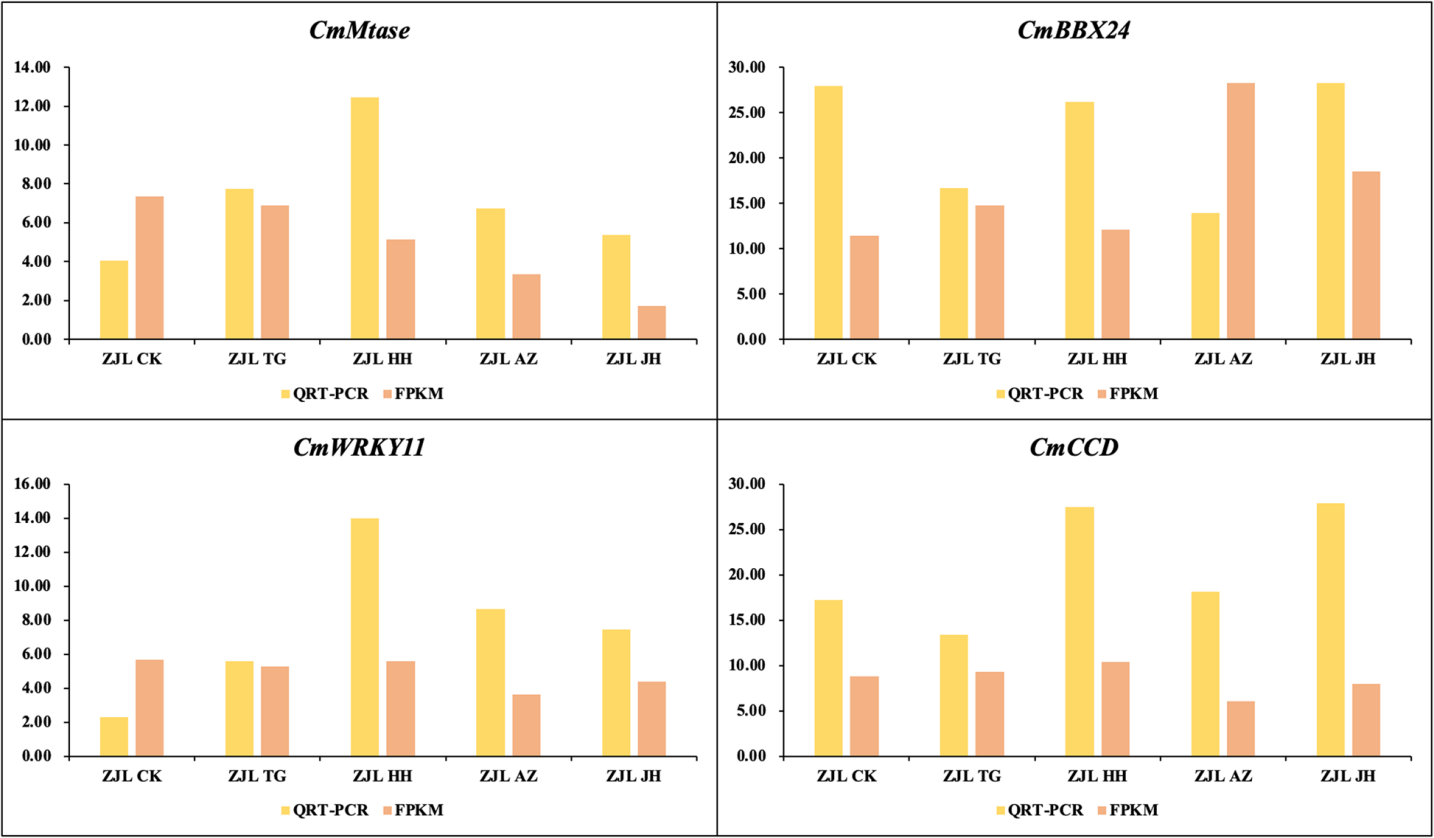
Verification of differently expressed genes using qRT-PCR. Note: The relative gene expression level was calculated by 2^-ΔΔCT^, and the expression level of ACTIN was the standard

Four DEGs were selected for gene expression verification by fluorescence quantitative PCR, which proved the accuracy of transcriptome sequencing. The fluorescent quantitative PCR results were consistent with the trend of the transcriptome expression profile (Fig. 11).

The DNA methyltransferase gene was annotated as Mtase, a gene that was differentially expressed. All treatments resulted in the downregulation of FPKM, and its expression decreased most in JH treatment, so both AZ and JH treatment might influence this gene. Surprisingly, fluorescence quantitative results showed that the expression level of HH treatment was higher than that of control, especially HH treatment, therefore, the role of this gene needs to be further studied and validated *CmBBX24* is associated with both photoperiod and Gibberellin (GA) biosynthesis pathways. *CmBBX24* is a flowering suppressor, and the downregulation of *CmBBX24* expression can advance flowering. The expression of the *CmBBX24* gene was not affected by HH treatment but increased by TG, AZ, and JH treatment. Our results are contrary to the results of this study, we hypothesized that the four treatments may not regulate flowering by affecting *CmBBX24* expression and thus the gibberellin pathway.

The FPKM of the *CmWRKY11* gene was slightly differentially expressed between different treatments, and all treatments increased the expression levels of *CmWRKY11* genes checked by qPCR with different amplitudes, indicating that *WRKY11* promotes flowering. Different treatments reduced the expression levels of flowering suppressor genes, thus promoting chrysanthemum flowering.

Carotenoid cleavage dioxygenases (CCD) are degradation enzymes involved in carotenoid metabolism in plants. Our results showed that there was little difference in the expression of the CCD gene between different treatments, and the qPCR results display that the expression of the CCD gene in HH and JH treatments was higher than that in the control and other treatments.

**Fig. 12:**
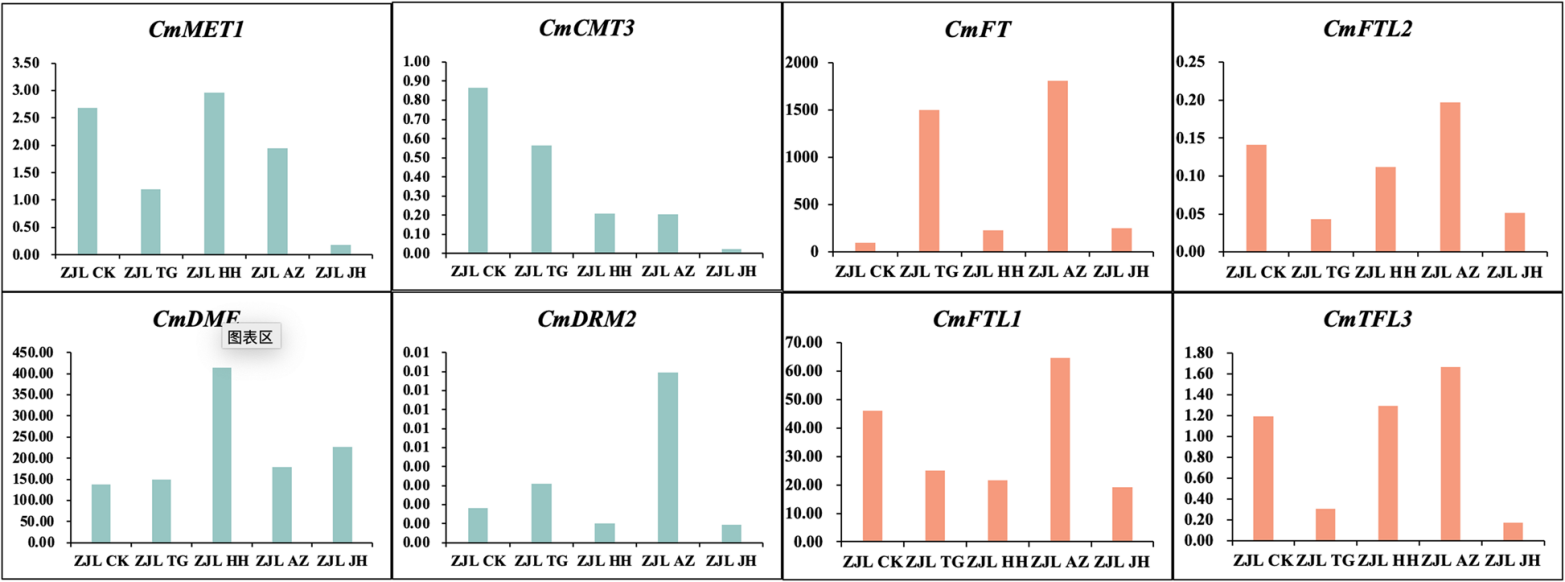
DNA methylation and flowering-related key gene expression using qRT-PCR. Note: The relative gene expression level was calculated by 2^-ΔΔCT^, and the expression level of ACTIN was the standard

We measured the expression levels of 4 DNA methylation transferase genes and found that HH treatment could not reduce *CmMET1* to the lowest extent, while curcumin treatment could reduce *CmMET1* expression to the greatest extent. AZ treatment could slightly reduce the *CmMET1* gene, but at a lower level than TG lines (Fig. 12). AZ treatment resulted in a significant upregulation of *CmDRM2*, while HH and JH inhibited *CmDRM2*, and *CmDME* gene was significantly upregulated only in HH treatment, while the other treatments had little effect. In *CmCMT3*, JH treatment decreased the most followed by AZ and HH while the reduction in TG was the least.

The expression of the FT gene was upregulated in all treatments, and significantly increased in TG and AZ lines, but less in JH and HH treatments. AZ treatment led to up-regulation of the expression levels of three FT-like genes, TG and JH treatment led to down-regulation of the expression levels of the best three genes, HH treatment led to down-regulation of *CmFTL1* and *CmFTL2*, and up-regulation of *CmFTL3*.

**Fig. 13:**
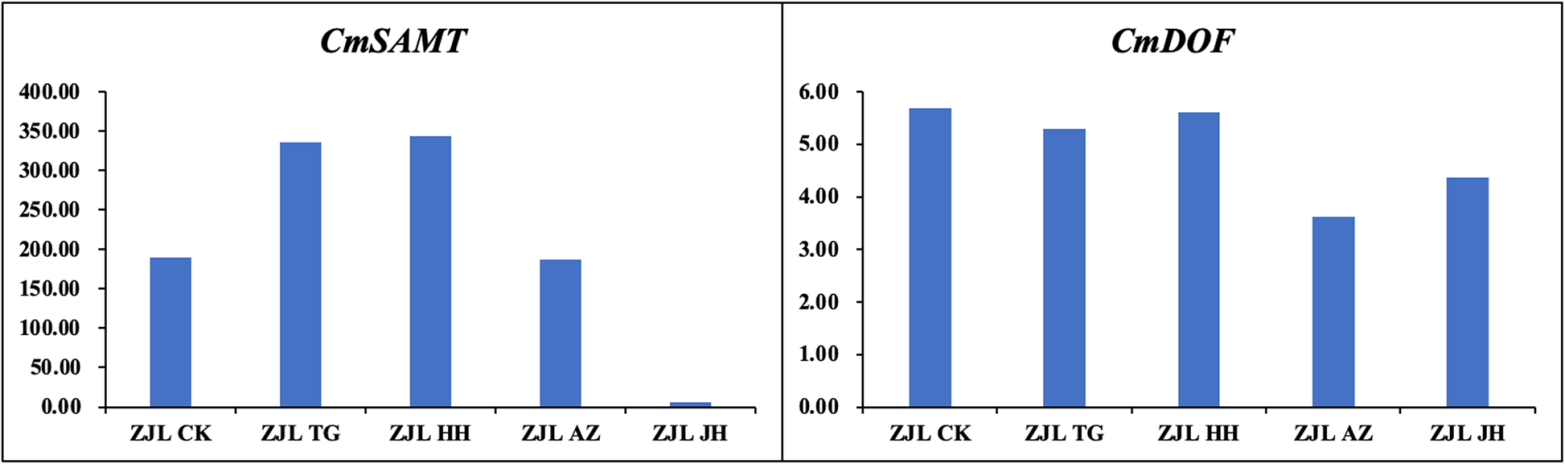
Gene expression validation of AZ and JH specifically affected gene using qRT-PCR. Note: The relative gene expression level was calculated by 2^-ΔΔCT^, and the expression level of ACTIN was the standard

*CmSAMT* gene was down-regulated by AZ and JH treatment, but up-regulated by HH and TG (Fig. 13). However, JH treatment resulted in the greatest downregulation, suggesting that the gene responsible was most affected by JH treatment. We screened a DOF transcription factor, and AZ and JH treatments led to the down-regulation of its expression, with the most down-regulation in response to AZ treatment. It was speculated that AZ treatment could specifically affect the expression of this transcription factor.

Different treatments had no consistent effect on the expression patterns of different genes, but mainly inhibited flowering suppressor genes and the DEGs suggested that the mechanism of early flowering might be related to transcription factors such as *WRKY* and *DOF* family members.

## Discussion

### Effects of DNA methylase inhibitors on gene expression

The main function of DNA methylase inhibitors is to inhibit the activity of related DNA methylation transferases in various ways, which can result in a change of gene DNA methylation state and expression level. These reagents can be applied to plant tissues or organs, resulting in differential gene expression and phenotypic variation. In vitro and in vivo laboratory models have shown that 5-azacytidine, one of the DNA methylation inhibitors, can inhibit hyper-methylation, restore suppressor gene expression, and have antitumor effects [31]. We used two reagents to explore their effects on plant genomes and analyzed the effects of different reagents. We found that the two reagents have different effects and could cause more differential gene expression compared with RNAi lines. However, no relevant research has been carried out on the changes in DNA methylation status in the genome, and future research will be carried out in this direction.

### Multiple DNA methylation-related genes cooperate to maintain genomic DNA methylation

There are four classes of DNA cytosine methyltransferases in plants: Methyltransferase (MET), Domains Rearranged Methyltransferase (DRM), DNA Nucleotide Methyltransferase 2 (DNMT2) and Chromomethylase (CMT, which is unique to plants) [32]. These genes play different roles in maintaining and reconstructing DNA methylation of genes.

While CMT3 catalyzes only maintenance methylation at CpHpG and CpHpH sites, MET1 catalyzes both maintenance and de novo methylation at CpG sites. DRM2 is related to the RdDM (RNA-directed DNA methylation) mechanism and catalyzes de novo methylation at CpG, CpHpG, and CpHpH sites [33–35]. The transcriptional activity of the DNA methylases/demethylases coding genes appeared to be correlated with the degree of genome DNA methylation. *MET1* catalyzes both maintenance and de novo methylation at CpG sites, while CMT3 catalyzes maintenance methylation at CpHpG and CpHpH sites. DRM2 catalyzes de novo methylation at CpG, CpHpG, and CpHpH sites, and is related to the RdDM (RNA-directed DNA methylation) mechanism [33–35]. The level of genome DNA methylation seemed to be correlated with the transcriptional activity of the coding genes of DNA methylases/demethylases.

The rolB gene transformation of *V. amurensis* cells reduced MET and CMT expression but significantly raised DRM expression. The 5-azaC treatment of the control and the rolB-transgenic calli significantly increased the expression of all methylases (excluding *MET*) [6]. The 5-azaC treatment caused a depletion in the DNA methylation levels in the promoter and coding regions of the VaSTS10 gene in both cell cultures [36]. A possible association between DNA hypomethylation and increased gene expression in the slmet1 mutants were revealed by transcriptome screening from tomato MET1 mutant lines 2,526 up DEGs. This experiment did not involve whole-genome sequencing, and further work will take into account defining the relationship between DEGs and CG site methylation status [37].

Our results showed that after 5-azaC treatment, the expression level of *CmMET1* was lowered to a level comparable to RNAi lines at the flowering stage. The function of *MET1* was to maintain the genomic DNA methylation state. It was speculated that 5-azaC treatment would cause the reduction in DNA methylation levels in the genome. There should be a close relationship between MET1 expression and the reduction of genomic methylation levels. However, there is no clear experimental evidence to prove the correlation between the two, which requires further exploration and research.

### WRKY transcription factors play a role in flowering regulation in chrysanthemum

As a plant-specific transcription factor family, the WRKY gene family is involved in multiple stages of plant development, mainly including flowering and stress response. *WRKY12*, *WRKY13,* and *WRKY71* genes play important roles in Arabidopsis flowering induction regulatory network [38–41].

The *WRKY* gene of chrysanthemum chamomile was identified and found to be overexpressed in several members of the *WRKY* transcription factor family during flowering induction and flower development. However, the specific mechanism remains unclear (unpublished). This time, the transcription factor family was analyzed in the transcriptome of chrysanthemum variety ZJL, and it was found that there were different expressions of multiple transcription factors, especially *WRKY* and *DOF* transcription factors. However, using transcriptome data, it was difficult to confirm the interaction characteristics of which members played an important role.

## Conclusion

This research adopts the AZ, JH, mixed processing, and MET1 silence strain 5 strains, etc. Flowering differences between the tested found multiple processing of the genomic DNA methylation level is reduced, so the transcriptome sequencing is carried out in the leaf of the flowering stage. The comparative analysis found that DEGs with AZ processing most, TG strain gene is less but more specific. Several flowering and DNA-related DEGs were expressed differently in MET1. Given that DNA methylation plays an important role in the regulation of the flowering stage, but there is no clear clue about specific regulatory genes, the DEGs obtained in this study provide gene resources and technical data for the subsequent development of related flowering stage regulation mechanisms.

## Materials and methods 

### Plant materials and treatment methods

In this study, the chrysanthemum variety ‘Zijingling’ used in this experiment is a multicolored, multi-headed cut chrysanthemum with purple ray florets. The control and transgenic strains (*MET1*_*-RNAi*_) of the chrysanthemum variety, ‘Zijingling’ (for short ZJL) were used as the research material to conduct relevant experiments. Details are provided in Table 4 for subcultures conducted over the same period and growing in a medium containing various DNA methylase inhibitors.

**Table 4:** Plant materials and treatment methods

**Strains**	**Abbreviations**	**DNA methylase inhibitors type and concentration**	**Medium**
Zijingling	ZJL CK	-	Hormone-free MS
Zijingling	ZJL AZ	5-azaC (100 μmol L^-1^)	Hormone-free MS
Zijingling	ZJL JH	Curcumin (200 μmol L^-1^)	Hormone-free MS
Zijingling	ZJL-HH	5-azaC (100 μmol L^-1^) & Curcumin (200 μmol L^-1^)	Hormone-free MS
MET1_-RNAi_	ZJL-TG	-	Hormone-free MS
MET1_-RNAi_	ZJL-TG&AZ	5-azaC (100 μmol L^-1^)	Hormone-free MS
MET1_-RNAi_	ZJL-TG&JH	Curcumin (200 μmol L^-1^)	Hormone-free MS
MET1_-RNAi_	ZJL-TG&HH	5-azaC (100 μmol L^-1^) &Curcumin (200 μmol L^-1^)	Hormone-free MS

The tissue culture material was grown in a dark period of 14h/10h at a temperature of 25±2°C and relative humidity of 70%. Tissue culture seedlings of chrysanthemum were rooted in MS medium for 30 days, and then the plants were removed. At this time, plant nutrient growth indices (plant height, root length, number of leaves, and number of roots) were measured.

After 30 days of treatment, the root culture medium of tissue culture seedlings was cleaned and transplanted into a 1:1 mixture of peat soil and vermiculite for growth. Flowering rates of different treatments were calculated at the early and middle flowering stages. At the mid-flowering stage, three plants for each treatment with consistent growth those were disease and pest-free, were selected. The top 3-5 leaves were collected at the same time and immediately frozen in liquid nitrogen and stored in a -80°C refrigerator for later use in whole-genome DNA extraction and transcriptome sequencing.

### DNA extraction, cDNA library construction, and sequencing

RNA isolation and purification from 15 samples were performed as previously described [22]. Preparation of the cDNA library and sequencing of 15 samples of *C. morifolium* for RNA-seq analysis were performed as described. In addition, a cDNA library for reference transcriptome sequencing was constructed using the RNA pool created from a combination of 15 RNA samples. The Illumina sequencing was conducted at Origin-gene Biomedical Technology Co., Ltd (Shanghai, China) according to the manufacturer’s instructions (Illumina Inc., San Diego, CA, USA). For RNA-seq analysis, a total of 15 sets of raw reads were obtained, corresponding to ZJL-CK-1, ZJL- CK-2, ZJL- CK-3, ZJL-AZ-1, ZJL-AZ-2, ZJL-AZ-3, ZJL-JH-1, ZJL-JH-2, ZJL-JH-3, ZJL-HH-1, ZJL-HH-2, ZJL-HH-3, ZJL-TG-1, ZJL-TG-2, and JL-TG-3.

### Genome-wide DNA methylation was detected by liquid chromatography

The DNA solution (50 μl) was mixed with 50 μl of 6 PA and incubated at 100 °C in a multiple-block heater (Model: K30, All sheng Instrument Co., Ltd., Hangzhou, China) for 80 min. After treatment at 37℃ for more than 30 min, 0.5 mol•L^-1^ EDTA was added to terminate the reaction. The supernatant was obtained after centrifugation at 1000 rpm and RT for 5 min. The pellets were washed with 100 μl of ultrapure water, and the supernatant was then separated via centrifugation. The two supernatants were pooled and used for HPLC analysis. The analysis parameters were set as follows: oven temperature at 37 °C, total flow of 1 ml, wavelength of 280 nm, injection volume of 10 μl. Mobile phase: 10% methanol, 7.0 mmol•L^-1^ sodium heptane sulfonate, 0.2% TAE; pH: 3.88; Flow rate: 0.5 mL min-1; Column: C18 (150×4.6mm, 5 μm). Linear equations of dC and 5mdC were established with different concentrations of standard substances. The linear equation of standard dC is Y = 4968.6x + 24229, R^2^ = 0.9986, the linear equation of standard 5 mdC is Y = 8980x-5100, R^2^ = 0.9936, the results showed that there is a good linear relationship between deoxycytidine and 5-methyldeoxycytidine in the range of 5-25 nmoL/mL. The DNA methylation level was expressed as a mole percent of 5mC in the sum of 5mC and C and calculated according to the equation: 5mC (%) = [5mC/ (5mC + C)] × 100%.

### Transcriptome data processing and analysis

Prior to downstream analysis, raw readings were cleaned to reduce data noise by eliminating the adaptor sequence, high concentrations of unknown bases, and low-quality reads. The genome of a nearby species of chrysanthemum, *C. nankingense* was utilized as the reference genome because C. morifolium lacked a suitable reference genome sequence. To obtain the annotations, the assembled unigene sequences were aligned using Blastn [23], Blastx [23], or Diamond [24] to the protein databases NR, Swiss-Prot, KEGG, and KOG, and InterProScan5 [25] to the protein database InterPro Scan5.GO annotations of unigenes were obtained using the Blast2 GO program together with the NR annotation. The estimated expression level was obtained using the FPKM technique. For each gene, FPKM comparisons were made between samples that had been exposed to different temperatures for the same number of days (CK versus AZ, CK vs JH, CK vs HH, CK vs TG, respectively). To identify the DEGs in the two samples, the method of Audic and Claverie was used [58]. Unigenes with a *P*-value of 0.01 and a |log2Ratio| were considered significant DEGs. All DEGs were assigned to each term of the GO and KEGG databases [26–28]. For the analysis of the transcription factors, |log2Ratio| > 2 was marked as significantly different between the samples.

### qRT-PCR analysis

The extraction of total RNA, synthesis of first-strand cDNA, and qRT-PCR was performed as previously described [10]. The primer sequences of the randomly chosen four DEGs are listed in Table S1. Other DNA methyltransferase, demethylase genes, and flowering genes are referred to in published papers [29]. The expression levels of candidate genes were normalized relative to the expression level of internal control of ACT [30] using the 2^-△△Ct^ method.

## Supplementary Information

**Additional file 1:** **Supplementary Figure 1.** Summary of the statistical table of transcript types. **Supplementary Figure 2.** The SNP point mutation analysis of 15 samples. **Supplemental Figure 3.** SNP comment location distribution analysis of 15 samples.

**Additional file 2:** **Supplementary Table 1.** Primers for PCR analysis. **Supplementary Table 2.** Database annotation statistics table. **Supplementary Table 3.** Top 30 differential pathways between 5 different groups. **Supplementary Table 4.** Top 10 differential pathways and detail between 5 different groups.

## Data Availability

The datasets used and/or analyzed during the current study are available from the corresponding author on reasonable request. All sequencing data have been uploaded to the NCBI Sequence Read Archive under SRA accessions: PRJNA747879.
