# Supplementary material for: Comparative analysis of the chrysanthemum transcriptome with DNA methylation inhibitors treatment and silencing MET1 lines
Source: BMC Plant Biol. 2023 Jan 21;23:47. doi: 10.1186/s12870-023-04036-x (PMC9862865; doi:10.1186/s12870-023-04036-x)
Supplement: Supplementary file 1 — Additional file 1: Supplementary Figure 1. Summary of the statistical table of transcript types. Supplementary Figure 2. The SNP point mutation analysis of 15 samples. Supplemental Figure 3. SNP comment location distribution analysis of 15 samples. [file 12870_2023_4036_MOESM1_ESM.docx]

Supplementary Figures


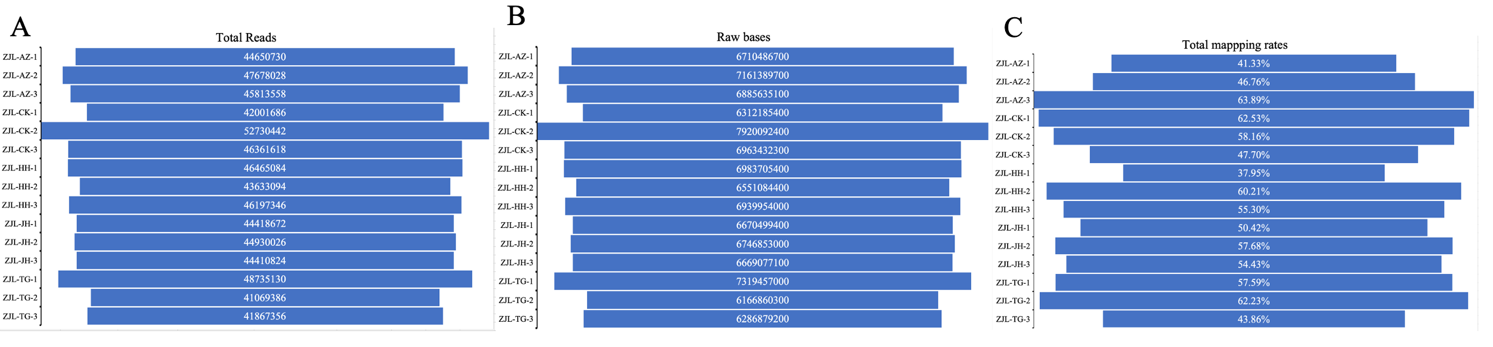


**Supplementary Figure 1.** Summary of the statistical table of transcript types


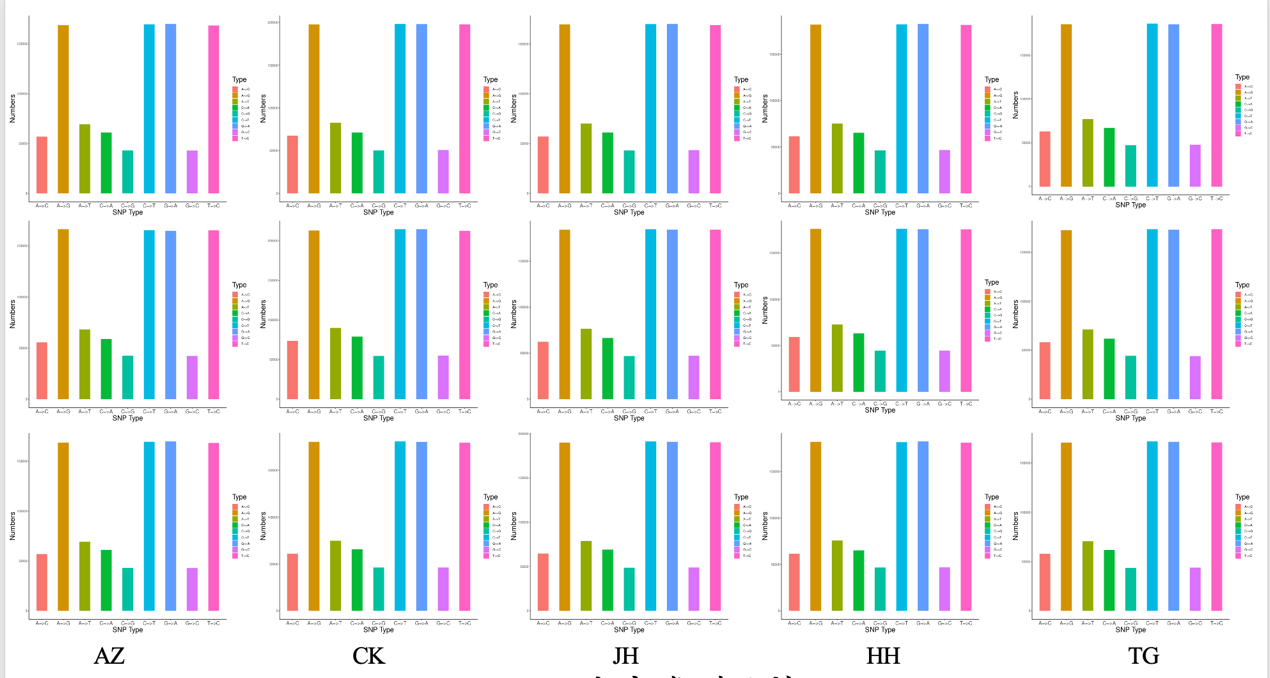


**Supplementary Figure 2.** The SNP point mutation analysis of 15 samples


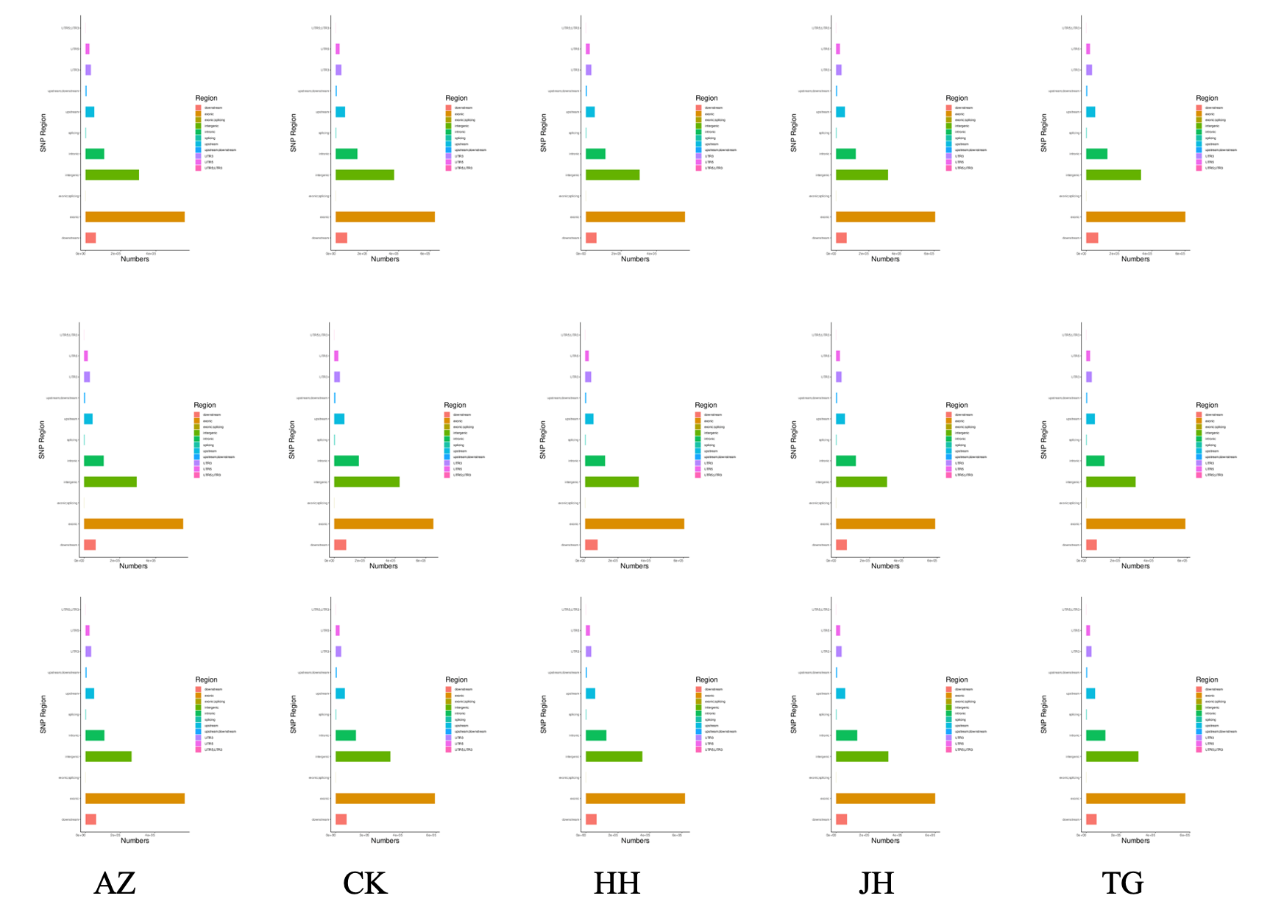


**Supplemental Figure 3.** SNP comment location distribution analysis of 15 samples
